# Pregnancy Outcome and Placenta Pathology in *Plasmodium berghei* ANKA Infected Mice Reproduce the Pathogenesis of Severe Malaria in Pregnant Women

**DOI:** 10.1371/journal.pone.0001608

**Published:** 2008-02-13

**Authors:** Rita Neres, Claudio R. F. Marinho, Lígia A. Gonçalves, Manuela Beirão Catarino, Carlos Penha-Gonçalves

**Affiliations:** 1 Instituto Gulbenkian de Ciência, Oeiras, Portugal; 2 Faculdade de Farmácia, Universidade de Lisboa, Lisboa, Portugal; Federal University of São Paulo, Brazil

## Abstract

Pregnancy-associated malaria (PAM) is expressed in a range of clinical complications that include increased disease severity in pregnant women, decreased fetal viability, intra-uterine growth retardation, low birth weight and infant mortality. The physiopathology of malaria in pregnancy is difficult to scrutinize and attempts were made in the past to use animal models for pregnancy malaria studies. Here, we describe a comprehensive mouse experimental model that recapitulates many of the pathological and clinical features typical of human severe malaria in pregnancy. We used *P. berghei* ANKA-GFP infection during pregnancy to evoke a prominent inflammatory response in the placenta that entails CD11b mononuclear infiltration, up-regulation of MIP-1 alpha chemokine and is associated with marked reduction of placental vascular spaces. Placenta pathology was associated with decreased fetal viability, intra-uterine growth retardation, gross post-natal growth impairment and increased disease severity in pregnant females. Moreover, we provide evidence that CSA and HA, known to mediate *P. falciparum* adhesion to human placenta, are also involved in mouse placental malaria infection. We propose that reduction of maternal blood flow in the placenta is a key pathogenic factor in murine pregnancy malaria and we hypothesize that exacerbated innate inflammatory responses to *Plasmodium* infected red blood cells trigger severe placenta pathology. This experimental model provides an opportunity to identify cell and molecular components of severe PAM pathogenesis and to investigate the inflammatory response that leads to the observed fetal and placental blood circulation abnormalities.

## Introduction

It is estimated that more than 50 million pregnancies occur every year in malaria endemic areas, and approximately half of these occur in sub-Saharan Africa, where *P. falciparum* transmission is most intense. Pregnancy-associated malaria (PAM) is one of the major public health problems in Africa with a high burden of maternal and fetal morbidity leading to 100,000 infant deaths per year [1], [2]. Pregnant women show increased malaria susceptibility and the severity of clinical manifestations are worse [3], both to the mother and fetuses, when maternal pre-immunition is inexistent or very low [4].

Together with maternal malaria induced anemia [5], [6], parasite sequestration in the placenta are thought to trigger a pathological process that contributes to decrease fetal viability and leads to infant Low Birth Weight (LBW) [7], [8], due to both preterm delivery and/or Intrauterine Growth Retardation (IUGR) [9], [10]. LBW (defined as birth weight <2500 g) is known to be the most important risk factor for infant mortality [11], [12]. The outcomes of PAM are influenced by different factors in different epidemiologic settings and are depending on the time of infection during the pregnancy period. In areas with a high rate of malaria transmission, infections in early pregnancy are associated with IUGR and abortions, whereas infections in later pregnancy are associated with preterm delivery [10], [13]. In contrast, pregnant women living in areas of low endemicity experience higher rates of abortion and stillbirth, associated to an elevated risk of maternal mortality [4], [13].

It is known that the severity of malaria is related to the capacity of *P. falciparum* infected Red Blood Cells (iRBC) to sequester in the microvasculature of vital organs. *Plasmodium falciparum* Erythrocyte Membrane Protein 1 (PfEMP1), a malarial variant antigen on infected erythrocytes, is involved in the adherence to host cell receptors. Infected erythrocytes can bind to endothelial receptors such as CD36 and intercellular adhesion molecule 1 (ICAM-1) [14]. However, studies on placental malaria have suggested that glycosaminoglycans (GAG) like chondroitin sulfate A (CSA) and hyaluronic acid (HA), play important roles as receptors for iRBC adhesion [13], [15], [16].

Although malaria in pregnancy has recently attracted many research efforts, there are ethical and logistic issues that restrict studies of human malaria infection during pregnancy and the specific pathologic bases of different PAM outcomes remain poorly understood [17]. Mouse malaria models have clear advantages for the study of PAM pathology due to the relative short gestational period that allows a reasonable experimental time frame and to the availability of a wide variety of immunological and genetic tools. In addition, recent reports provided detailed anatomy and physiology analysis of mouse placenta [18]–[20], a key organ in PAM pathogenesis and pointed out considerable sharing of cell and molecular features with the human placenta, including the hemochorial barrier and the maternal antibody transmission to the fetus across the placenta [20]–[23].

Previous studies concerning PAM in rodents have focused in congenital malaria [24] and in the characterization of placenta pathology [25], [26]. Other studies of rodent malaria in pregnancy aimed to observe disease dynamics and recrudescence [27]–[32] and to analyze pregnancy outcome upon disease drug treatment [33]. A recent report described disturbances in mouse pregnancy outcome after *P. chabaudi* infection but did not present pathology comparable to human placental malaria evoked by *P. falciparum* [34]. Despite these attempts, a thorough characterization of a mouse model reproducing the main features of severe malaria in pregnancy has not been described.

We revisited the study of severe placenta malaria pathology in the mouse through an experimental model that uses *P. berghei* ANKA-GFP to infect BALB/c mice during pregnancy, resulting in placenta damage and inflammation, as well as IUGR/LBW. In addition, we found that *P. berghei* ANKA-GFP iRBC are able to bind to receptors present in mouse placental tissues providing the basis for a pathology trigger of mouse placenta pathology, comparable to the mechanism proposed for human placental malaria.

This experimental model captures many pathology features analogous to severe malaria manifestations in pregnant women and may provide opportunities to investigate the pathogenesis mechanisms of malaria in pregnancy and enable experimental evaluation of PAM interventions strategies.

## Materials and Methods

### Animals and parasites

The BALB/c mice were bred and maintained in conventional housing at the Instituto Gulbenkian de Ciência. Infection experiments were performed in adult females, between 10–15 weeks of age. *P. berghei* ANKA constitutively expressing green fluorescent protein (*P. berghei* ANKA-GFP) (15cy1 clone) [35], [36] was kindly provided by Professor Andrew P. Waters (Leiden University Medical Center, Leiden, The Netherlands). Infected red blood cells (iRBC) used in experimental infections were obtained from *in vivo* passage in BALB/c mice, when the percentage of iRBC reached approximately 10%. Parasitemia was measured using flow cytometry analysis as described elsewhere [37]. All animals were fed with regular diet and all procedures were in accordance with national regulations on animal experimentation and welfare and were authorized by the Instituto Gulbenkian de Ciência animal welfare committee.

### Gestation timing and pregnancy monitoring

Detection of the vaginal plug and measurement of body weight were jointly used to time gestation, as described by Freyre et al. [38]. Two to three females were put together with one male for 2 days, and examined for the presence of vaginal plug every morning. The day of finding the vaginal plug was considered as gestation day one (G1) and pregnancy progression was monitored every other day by weighting the females. Successful fertilization was confirmed between G10 and G13 when the animals had an average increase of 3–4 g in body weight. Thus, weight gain was taken as sign of pregnancy and abrupt weight loss as indicator of pregnancy damage or interruption.

### Pregnancy experimental infection

Pregnant mice were intravenously (IV) infected between G11 and G13 with 10^6^ iRBC, and parasitemia was recorded every other day. To evidence pathological features of malaria during the course of pregnancy and in the developing fetus, this infection period was determined to be the optimal time point as earlier infections did not allow reaching pregnancy at term (data not shown), which is consistent with previous reports [26], [27], [31]. Non-pregnant infected females or non-infected pregnant females were used as controls in pregnancy infection experiments as appropriate. Part of the pregnant females (both infected and controls) were allowed to deliver and the progenies were followed up to weaning. The other pregnant females were subjected to caesarian section between G17 and G19 for fetal survival and placenta pathology observation.

### Offspring monitoring

As *P. berghei* ANKA-GFP infection is lethal in BALB/c mice, foster mothers were used in newborn post-natal follow-up studies. Hence, both newborns from infected mothers and newborns from control mothers were also transferred to foster mothers to avoid weight bias due to differential maternal nourishment. The newborns were weighted every other day.

### Fetal survival evaluation

Between G17 and G19, pregnant females were killed by CO_2_ narcosis, their spleens were weighted, the uterus was examined and the number of fetuses and resorptions were recorded. Resorptions were identified as small implants with no discernible fetus and placenta, corresponding to embryos that died before complete placenta vascularization. The fetuses were extracted from their amniotic envelop and viability was immediately evaluated by prompted movement reaction to touching with pliers. The lack of reactive movement indicated that the fetus had recently died and was considered an abortion. Macerated pale white fetuses were dead and were also recorded as abortions. Fetuses and placentas were separately weighted. Non-aborted fetuses were killed combining CO_2_ narcosis and hypothermia.

### Tissue preparation and histopathological analysis

Placentas from infected and non-infected females were treated in a similar way. Placentas were separated in two halves, one half was fixed in 1.6% paraformaldehyde with 20% sucrose for further processing and the other half collected for RNA extraction. Paraffin-embedded non-consecutive placenta sections were stained with hematoxylin-eosin (HE) and examined under a light microscope (Leica DM LB2, Leica Microsystems). For histological and morphometric analysis, placental sections were examined in a blind fashion.

### Immunohistochemistry

Fixed placenta samples were washed in PBS with 15% sucrose overnight, soaked in Tissue-Tek® (Sakura) and frozen in dry ice. For immunohistochemistry staining, freshly made frozen sections (6 μm thick) were rinsed in PBS for 30 minutes and blocked with 1% bovine serum albumin (BSA). To enhance parasite GFP signal, we used rabbit polyclonal anti-GFP antibody (Molecular Probes) and goat anti-rabbit antibody conjugated with Alexa488 (Molecular Probes). To identify macrophages/monocytes we used anti-CD11b biotinilated antibodies (BD Biosciences, Pharmingen), followed by incubation with Rhodamin-Avidin D (Vector Laboratories). Nuclei were stained with DAPI (Invitrogen) and coverslips were mounted with aqueous mounting media (Mowiol 4-88, Calbiochem). Stained sections were examined under fluorescence microscopy (Leica DMRA2, Leica Microsystems).

### Morphometric analysis

HE stained placental sections were analyzed for vascular space quantification. In each section, 5 randomly selected microscopic fields in the labyrinthine region (magnification ×40) were acquired at 1280×960 resolution, using a color video camera (Evolution™ MP color, Media Cybernetics) connected to a light microscope (Leica DM LB2, Leica Microsystems). We implemented an image analysis routine using ImageJ (ImageJ 1.37v, National Institutes of Health). Briefly, after acquisition, the images underwent an automated light analysis procedure where noise removal was applied to ensure color and image quality standardization across sections and specimens. The images were given a color threshold to cover the area corresponding to blood spaces lumen. The coverage percentage was calculated as the ratio between the number of pixels covered by the area defined by the threshold and the overall number of pixels in the image. The blood vascular area in each placenta was estimated from the analysis of two non-consecutive sections. The reported results correspond to individual pregnant females and represent the average result for 2-3 placentas.

### RNA isolation and chemokine gene expression

Total RNA from freshly collected placentas was obtained using an RNeasy Mini Kit (Qiagen), following the manufacturer protocol for animal tissues. One microgram of total RNA was converted to cDNA (Transcriptor First Strand cDNA Synthesis Kit, Roche) using random hexamer primers. MCP-1 (CCL2) and MIP-1 alpha (CCL3) expression was quantified using TaqMan Gene Expression Assays from ABI (Mm00441242_m1 and Mm00441258_m1, respectively) with TaqMan Universal PCR master mix. The gene expression quantification reactions were performed in an ABI Prism 7900HT system. Relative quantification of MCP-1 (CCL2) and MIP-1 alpha (CCL3) in each real-time PCR reaction was obtained after normalization for GAPDH expression measured in the same PCR reaction.

### Synchronization of parasitized erythrocytes

iRBC were collected from infected animals with 10–20% parasitemia. In order to obtain mature blood stage parasite forms (trophozoites/schizonts), *P. berghei* ANKA-GFP infected erythrocytes were synchronized as described elsewhere [39]. After mature forms enrichment, infected erythrocytes were resuspended in 1% BSA in PBS at a concentration of 10^8^ iRBC/ml.

### Cytoadherence assays

Placentas from uninfected BALB/c females, obtained at G19, were treated using a previously described protocol [40]. Briefly, the placentas were fixed in 2% formalin and 0.5% glutaraldehyde for 10 minutes, heated in a microwave oven before being paraffin-embeded, and cut into sections of 5 µm onto glass slides. This fixation protocol aims to preserves the binding capacity of glycosylaminoglycans (GAG) in the placenta intervillous spaces [40]. Tissue sections, after deparaffinized and rehydrated, were delimitated with a DAKO pen. For placenta-receptor cleavage experiments, placental sections were incubated with 0.5 U/ml chondroitinase ABC (from *Proteus vulgaris*, Sigma), with 30 µg/ml hyaluronidase (from bovine tests, Sigma), with heparinase II (from *Flavobacterium heparinum*, Sigma) or with PBS for 2 periods of 2 hours at 37°C. Both enzyme-treated sections and non-treated sections were blocked with 1% BSA in PBS at room temperature for 30 minutes. Fifty microliters of synchronized iRBC suspension, at the concentration of 10^8^/ml, were overlaid onto each tissue section for 60 minutes at 37°C in a humid chamber. After washing the unbound cells, the placental sections were incubated with DAPI. For iRBC-ligand blocking experiments, synchronized iRBC were preincubated with the indicated concentrations of chondroitin sulfate A (CSA) from bovine trachea (Sigma), hyaluronic acid (HA) potassium salt from human umbilical cord (Sigma) or colominic acid sodium salt (as negative control) from *E. coli* (Sigma), at 37°C for 30 minutes with moderate agitation. For iRBC-ligand cleavage assays iRBC were treated with trypsin (Gibco), proteinase K (Sigma) or neuraminidase as a negative control (from *Clostridium perfringens*, Sigma). iRBC were pre-incubated with each enzyme at indicated concentrations for 30 minutes at 37°C. After washing, iRBC were overlaid on placental sections as described above. The slides were mounted with Mowiol and examined under fluorescence microscopy (magnification ×40). The number of iRBC adhering placental sections in each experimental condition was determined in a blind fashion, counting 50 fields in each of 3 independent experiments.

### Statistical analysis

Statistical differences between groups of mice used in this study were evaluated by the Student's t test. Mann-Whitney test was used for morphometric data and Log Rank test for survival curves.

## Results

### Experimental model

Our search for an experimental model that recapitulates typical pathology features of severe malaria in pregnancy revealed that time of infection during pregnancy is critical to evidence poor pregnancy outcome and fetal growth impairments. In fact, infection at early stages led to premature pregnancy interruption while infection at G13 allowed pregnancy to proceed to later stages when fetal and placenta pathology became apparent and resembled human PAM. Moreover, such pathological features were exuberant in the experimental model here reported, which used lethal *P. berghei-*GFP to infect BALB/c female mice that are resistant to cerebral malaria, allowing progression of the disease to hyperparasitemia states. Other parasite species/mouse strains combinations were tested and we noted that *P. chabaudi* did not elicit pronounced signs of placental malaria and C57Bl/6 and DBA/2 mouse strains could not survive to *P. berghei-*GFP infection long enough to exhibit typical manifestations of malaria in pregnancy (data not shown).

### Increased malaria susceptibility in pregnant mice

**Figure 1:**
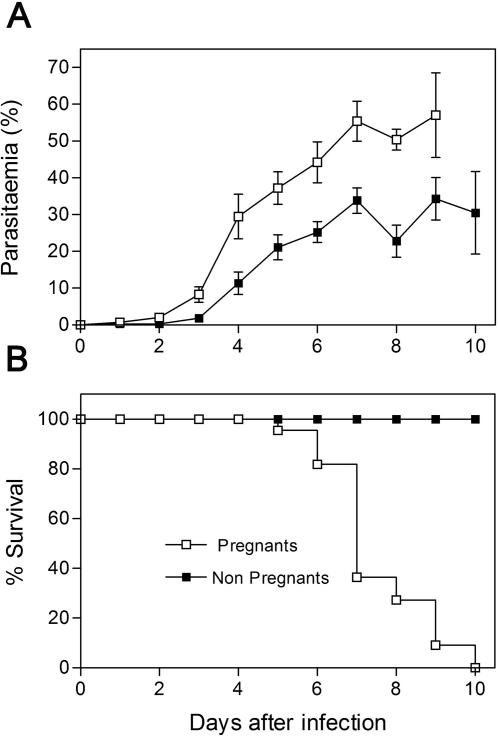
Increased disease susceptibility in pregnant BALB/c mice infected with *P. berghei-*GFP. BALB/c pregnant females were infected on G13 by IV injection of 10^6^ iRBC and non-pregnant females were simultaneously infected. The plots represent cumulative results of three independent experiments in a total of 32 pregnant and 16 non-pregnant females. (A) Parasitemia curves where data points represent mean±SE. From day 3 post-infection onwards parasitemia was significantly higher in pregnant females (*P-*value<0.05). (B) Survival curves up to 10 days after infection show that survival time of pregnant female mice are significantly lower than in controls (*P*-value<0.0001). It should be noted that non-pregnant females died at a later stage with hyperparasitemia.

Comparison of *P. berghei-*GFP course of infection in pregnant and non-pregnant females confirmed earlier findings that pregnancy in mice confers an increased susceptibility to malaria [26], [27], [33] and showed that pregnant mice experienced faster increase in parasitemia as compared to non-pregnant females. Parasitemia in pregnant mice was 55.41±5.44% (mean±SE) on day 7 post-infection as compared to 33.83±3.47% in non-pregnant mice (*p* = 0.007) (Figure 1A). In addition, survival to infection was reduced in pregnant mice, with all deaths occurring between day 5 and day 10 post-infection (Figure 1B). In contrast, the majority of non-pregnant infected females survived until day 20 post-infection and by day 30 all had succumbed to infection (data not shown). Average survival time for pregnant and non-pregnant infected mice was 7.5 and 20.5 days, respectively. These results suggest that, similarly to humans, pregnant mice show increased susceptibility to malaria infection which may affect their progeny or compromise pregnancy.

### Unsuccessful pregnancy and impaired post-natal growth

We followed-up pregnancy outcome in 22 infected females and found out that malaria had a strong negative effect in pregnancy success (Table 1). Approximately two-thirds of infected pregnant females (14 out of 22) did not give rise to viable pups due to maternal death before parturition (8 cases) or to preterm delivery/abortions (6 cases). The remaining mothers carried out pregnancy to term giving rise to 27 viable newborns. The progeny of 2 infected mothers, out of 8 that gave birth, died after birth between day 2 and day 21 (data not shown), indicating that malaria during pregnancy increases pups mortality.

**Table 1:** Effect of *Plasmodium berghei* infection during pregnancy on reproductive outcome and fetus development (a)

*P. berghei-*GFP exposure	No. of pregnants	Gestational period (days)(b)	Birth weight (g)(b)	Weight day10 (g)(b)	No. Successful fetus(b)	No. Unsuccessful pregnancies(c)
Infected	22	19.8	1.3	3.4	5	14(8/6)
Uninfected	14	20.7	1.4	5.6	6	0
*p*- value(d)	__	0.05	0.03	<0.0001	0.39	__

(a) BALB/c mothers were infected on G13 with *P.berghei* by IV injection of 10^6^ iRBC and were allowed to give birth at term.

(b) Average values.

(c) Number of unsuccessful pregnancies (mother dead pregnant/preterm delivery or abortion).

(d) Student's *t* test.

**Figure 2:**
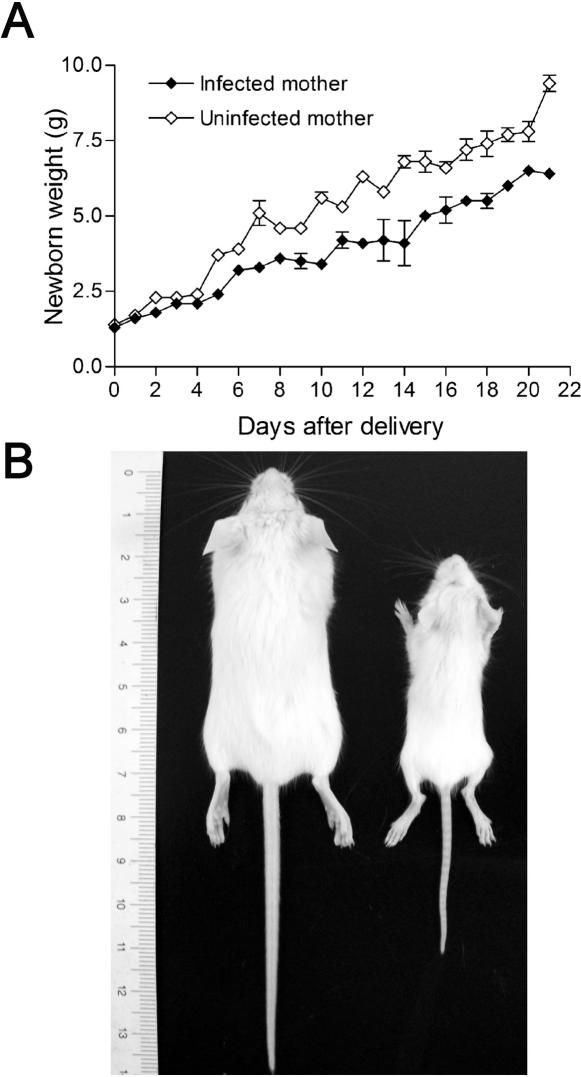
Reduced growth rate in progenies of *P. berghei-*GFP infected mothers. BALB/c pregnant females were infected on G13 by IV injection of 10^6^ iRBC. After delivery newborns were transferred to a foster mother and their body weight was followed up to weaning (A). Example of body size difference at day 21 of age is shown in (B), mouse born from non-infected (left side) and from infected mother (right side).

Post-natal growth of viable newborns was followed up to weaning by weight monitoring and compared to 49 newborns from non-infected mothers. In this experiment, infected mothers would not survive long enough to nourish their pups and we used foster mothers to feed all newborns as described in the methods section. Low growth rate was observed in the majority of the newborns from infected mothers (Figure 2A) resulting in obvious body size discrepancy (Figure 2B). At day 10 after birth pups born from infected mothers weighted significantly less (3.4±0.16 g) than pups born from control mice (5.6±0.19 g) (Table 1), suggesting that the growth impairment in the first stages of post-natal life may result from intrauterine fetal growth impairment. Nevertheless, those mice were able to develop until adulthood when they were apparently normal (data not shown).

### Fetal survival and intrauterine growth retardation

**Figure 3:**
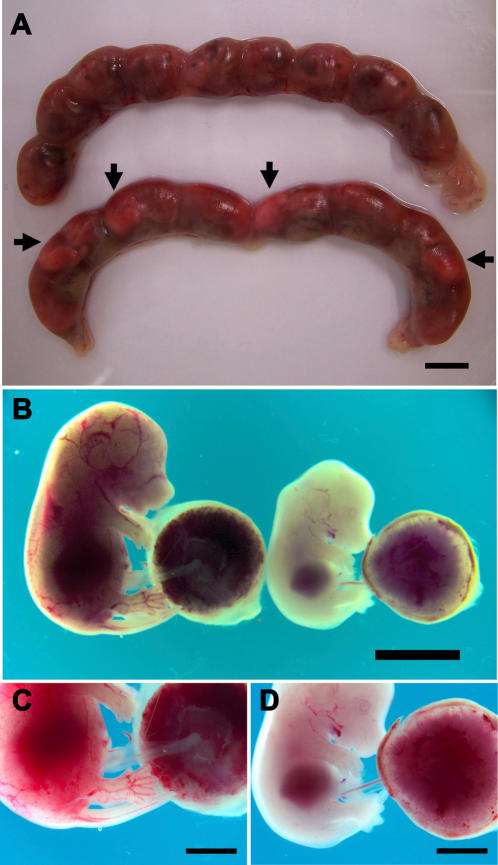
* P. berghei-*GFP infection impairs pregnancy outcome and fetus development. (A) Representative uterus at G18 from BALB/c pregnant females, uninfected (upper) and infected on G13 with *P. berghei-*GFP by IV injection of 10^6^ iRBC (bottom). The arrowheads indicate abortions. (B) Fetus from uninfected (left) and from infected mother (right). In detail, mouse placenta from an uninfected (C) and infected mother (D). Lack of blood circulation is noticeable in the placenta, paws and tail in panel (D). Scale bar represents 1 cm in A–B and 0.5 cm in C–D.

We evaluated the effects of malaria in pregnancy on fetal survival and fetal growth at late pregnancy stages (G18) by analyzing fetuses from 28 pregnant females infected at G13 and from 9 non-infected pregnant females (Table 2). Uterus collected at G18 from infected pregnancies frequently showed macroscopic abnormalities, as compared to controls, corresponding to the presence of aborted fetuses (Figure 3A). In fact, infected mothers had significantly lower number of viable fetuses as compared to non-infected mothers (*p* = 0.01) and had higher number of aborted fetuses (*p* = 0.002) (Table 2).

**Table 2:** Pregnancy outcome obtained at caesarean section on G18 after *Plasmodium berghei* infection during pregnancy(a)

*P. berghei*-GFP exposure	No. of pregnants(b)	Mother's spleen weight (mg)(c)	Fetus weight (g)(c)	No. Abortions(c)	No. Resorptions(c)	No. Successful fetus(c)
Infected	28	521	0.6	2.4	1.7	3.7
Uninfected	9	102	1.0	0.2	0.7	7.7
p-value(d)	__	0.02	<0.0001	0.002	0.11	0.01

(a) BALB/c mothers were infected G13 with *P.berghei* by IV injection of 10^6^ iRBC.

(b) Pregnants sacrificed at G18.

(c) Average values.

(d) Student's *t* test.

We searched for signs of fetal impaired development in the uterus. Fetuses from uninfected healthy mothers showed pink coloration, had translucent skin with visible blood flow in the blood vessels and the placentas were replenished with blood (Figure 3B left and C). In contrast, many fetuses from infected mothers appeared abnormal having remarkable reduced size, pale tone with poor blood vessel replenishment and placentas with reduced blood content (Figure 3B right and D). It is worth to remark (Table 2) that average weight of viable fetus at G18 was significantly lower in infected mothers (0.55±0.034 g) as compared to non-infected mothers (0.9±0.053 g). Together, these data strongly suggest that fetuses from infected mothers suffer IUGR and have decreased viability due to placenta blood flow impairments, recapitulating pathological features of severe malaria manifestations typically observed in pregnant women from low malaria transmission regions [4].

### Placenta inflammation

**Figure 4:**
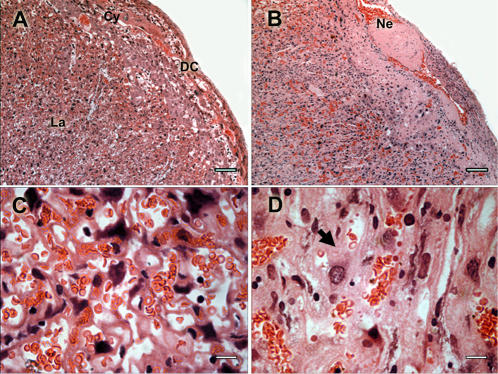
Placenta pathology in infected pregnant mice. Histology of infected placentas collected at G18. HE stained sections from non-infected mice (panels A and C) and infected (panels B and D) are depicted. Different cell types are identified in panel A as (DC) decidual cells, (Cy) cytotrophoblastic cells and (La) labyrinthic cells. Fibrinoid necrosis areas (Ne) are indicated in panel B. Arrowhead in D shows syncytiotrophoblast tissue thickening. Scale bar represents 100 μm in (A–B), and 10 μm in (C–D).

**Figure 5:**
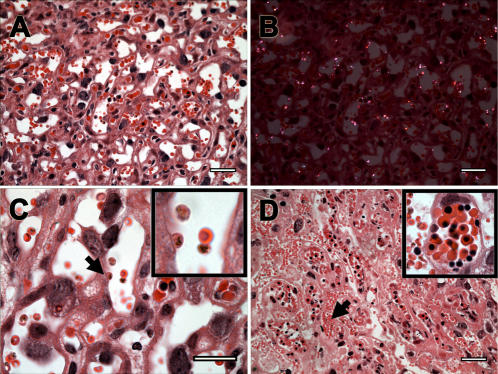
Placental malaria. HE stained placentas from BALB/c females infected with *P. berghei*-GFP and collected at G18. (A) Image from severely infected placenta with high number of parasitized maternal erythrocytes. (B) The same field as (A) under polarization microscopy revealing hemozoin parasite pigment. (C) Arrowhead and insert show an infected erythrocyte adhered to the syncytiotrophoblast layer. (D) Placental section with infected erythrocytes (arrowhead) in the maternal blood and fetal erythroblasts that were enhanced in the insert. Scale bar represents 30 μm in (A, B and D) and 20 μm in (C).

**Figure 6:**
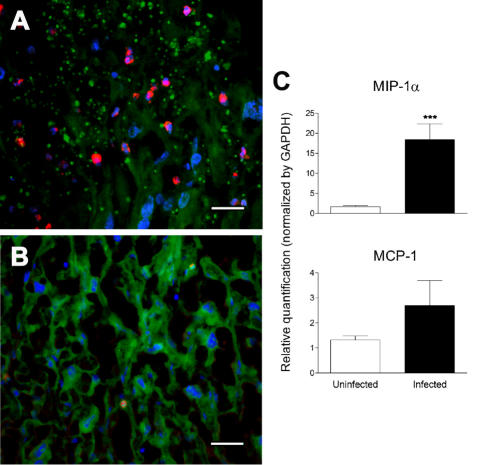
Inflammatory infiltration and macrophage/monocyte attractant chemokine expression in malaria infected placenta. (A) Immunohistochemistry analysis of placentas from BALB/c females infected on G13 with *P. berghei*-GFP iRBC and collected at G18 that were stained with anti-GFP (green) and anti-CD11b (red) revealing the presence of parasites on vascular walls and monocytes/macrophages infiltration, respectively. The (B) panel represents sections of non infected placentas. The cell nuclei were stained with DAPI (blue). Scale bar represents 30 μm. (C) RNA expression of MIP-1 alpha and MCP-1 genes were quantified in 30 infected and 8 uninfected BALB/c placentas collected on G18. Relative quantification was obtained by normalization for GAPDH expression. Each bar represents the mean±standard error of individual values. P-value = 0.0002 is represented by ***.

Placenta represents the interface between mother and fetus, playing a critical role in fetal growth and development. Placental tissue of infected pregnant females revealed a number of abnormalities in comparison to non-infected controls (Figure 4). We repeatedly observed significant thickening and disorganization of labyrinthine zone, distension and disarrangements of perivascular space (Figure 4D), as well as presence of parasitized red blood cells in the maternal blood space (Figure 5A–C). Hemozoin, a malaria parasite pigment, was observed in most of the infected placentas (Figure 5B). Fetal blood circulation often contained larger amount of erythroblasts (Figure 5D) but it never presented any sign of parasites or hemozoin. Some specimens showed focal fibrinoid necrosis in the placenta basal zone (Figure 4B), hyperplasia of syncytiotrophoblastic cells (Figure 4D) and accumulation of mononuclear cells in the maternal blood space revealed by immunofluorescence staining. We evidenced accumulation of CD11b expressing cells (Figure 6A), indicating that the infiltrate was predominantly composed by monocytes/macrophages. This result prompted us to measure the expression of macrophages attracting chemokines MIP-1 alpha and MCP-1 in the placenta. RNA quantification revealed that MIP-1 alpha gene expression was significantly increased in the infected placenta (Figure 6C) providing support for the notion that cell and molecular components of the innate immune system participate in the host response to the placenta malaria infection.

### Placental vascular space impairment

**Figure 7:**
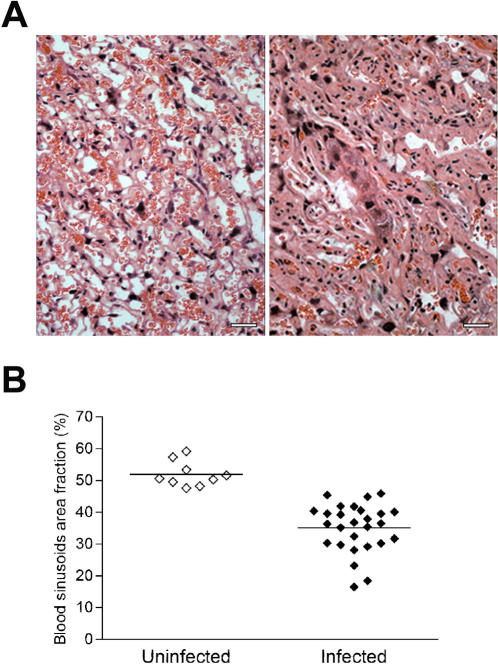
Reduction of placental vascular space in infected pregnant mice. (A) The available area for blood circulation at G18 is reduced in the case of infected placentas (A, right) in comparison with non-infected (A, left). Scale bar represents 25 μm. (B) The placental area occupied by blood sinusoids was quantified in relation to the total placental area using an automated morphometric procedure, as described in Materials and Methods (*p*<0.001, Mann Whitney Test).

The alterations in tissue organization observed in the infected placenta suggested that the maternal blood flow could be reduced in pregnancy malaria. Thus, we used a computerized morphometry method to quantify cross-sectional areas of blood sinusoids in placental labyrinthine region. Morphometric analysis was performed as described in methods section and confirmed that the blood sinusoids areas differed significantly between infected and non-infected placentas. The average blood sinusoid area was 52.0±4.0 (mean±SD, arbitrary units) in the control group and it dropped to 34.7±7.5 (*p*<0.0001) in the infected pregnant group (Figure 7). The blood sinusoids area was measured in five different regions of the labyrinthine zone and in all of them the area decreased in similar degree, indicating that this phenomenon is spread across the placenta rather than restricted to specific areas. Together, the data suggest that alterations of pregnancy outcomes observed in mice infected with *P*. *berghei*-GFP correlate with pathological alterations of the placenta tissue, involving inflammation, tissue disorganization, reduction of vascular spaces and consequent reduction in blood supply.

### iRBC binding to placental sections

**Figure 8:**
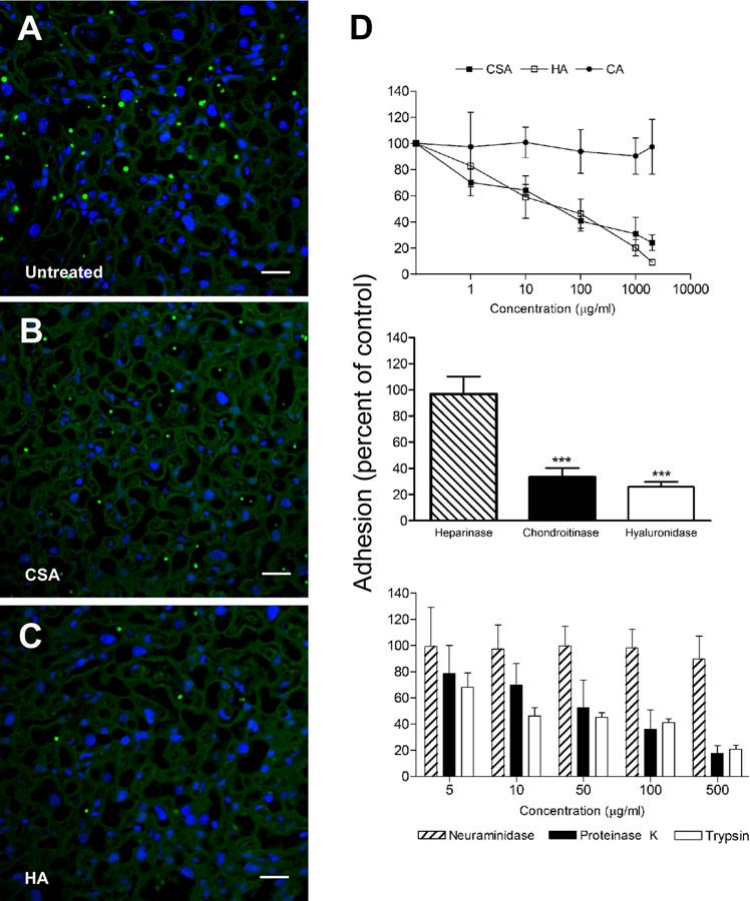
* Ex vivo* adherence of *P. berghei*-GFP iRBC to mouse placenta. (A) Typical microscopic image of adherence assays showing iRBC adhered in the intervillous space and to syncytiotrophoblast cell layer (A). Representative images of blocking adherence assays where iRBC were pre-incubated with 2 mg/ml of CSA (B) and HA (C). IRBC were preincubated with increasing concentrations of HA, CSA and colominic acid and then used in binding assays as described in materials and methods (D, upper graph). Adhesion of iRBC to uninfected placental tissue pre-treated with chondroitinase ABC and hyaluronidase but not with heparinase (D, middle graph). Intact iRBC were treated with neuraminidase, proteinase K and trypsin prior incubation with the placental tissue (D, lower graph). All data represent the proportion of bound iRBC expressed as a percentage of control (mean±s.e.m. for three experiments). *P*-value<0.001 is represented by ***.

Several receptors in human placenta have been suggested to mediate *P. falciparum* cytoadhesion and sequestration and we investigated the involvement of chondroitin sulfate A (CSA) and hyaluronic acid (HA) in *P. berghei-*GFP iRBC cytoadhesion. Using a set of *ex vivo* adhesion assays, we verified that CSA and/or HA are involved in specific interactions of *P. berghei*-GFP blood stages with the placental tissue (Figure 8). In fact, iRBC adherence was significantly reduced if parasite mature forms were previously incubated with CSA (Figure 8B) or HA (Figure 8C). Furthermore, adhesion was competitively inhibited in a dose-dependent fashion by both CSA (69% reduction at 1mg/ml) and HA (80% reduction at 1mg/ml) , but not with colominic acid (Figure 8D, upper). In addition, iRBC adherence also registered a significant reduction on tissue sections pre-treated with chondroitinase (66% reduction) or hyaluronidase (74% reduction), but heparinase had no effect on the iRBC adhesion (Figure 8D, middle). With the aim of demonstrating that adhesion properties of iRBC were dependent on surface proteins, we pre-treated iRBC with two proteolytic enzymes (trypsin and proteinase K) and a non-proteolic control enzyme (neuraminidase). Proteolitic depletion of iRBC surface proteins showed to reduce adhesion capacity in a concentration-dependent manner (Figure 8D, bottom). These results demonstrate that *P. berghei* iRBC adhesion is partially dependent on the presence of CSA and HA receptors in the placenta and is inhibited by blocking their putative ligands in *P. berghei-*GFP iRBC. These findings strongly suggest that CSA and HA in the mouse placental tissue participate in adhesion of iRBC and may be involved in parasite sequestration and in the consequent triggering the placenta pathology events associated to malaria.

## Discussion

Here we described pathological manifestations resembling human malaria in pregnancy that were identified in a mouse model that uses *P. berghei*-GFP. Accordingly to Desowitz [23], the attributes of an experimental model of malaria in pregnancy should account for superior parasitemia and virulence for the mothers, when compared to non pregnant controls, and harmful consequences for the fetuses. Preferably, such an experimental model would show reasonable pathogenesis congruency to human disease characteristics particularly in relation to placental sequestration/cytoadherence properties. We showed that infecting BALB/c mice with *P. berghei*-GFP during pregnancy results in increased disease severity and impairments in fetal viability and post-natal growth. These findings compel comparisons to the placental damage and inflammation that are underlying the clinical manifestations observed in humans [5]–[10]. Moreover, cytoadherence assays support the hypothesis that *P. berghei*-GFP iRBC encounter placental receptors that promote specific binding in analogy to the proposed placenta sequestration mechanism for *P. falciparum* [40], [41].

In areas where malaria transmission is low or unstable, the levels of immunization are weak or inexistent and PAM clinical outcomes seem to be more severe for both to the mother and fetus. The experimental model here presented was established in non-immune mice, which enhanced disease severity and magnified pathology phenotypes as compared to the human disease. Such phenotypic exacerbation might be advantageous in identifying molecular and cell host components playing key roles in the pathogenesis mechanisms, leading to the different pathology features observed in this experimental model of severe malaria in pregnancy. Although *P. falciparum* infection in pregnant women leads to lower parasitemia and is usually less severe as compared to the mouse phenotypes here reported, it is worth mentioning that primigravida women have been suggested to be more susceptible to severe malaria in pregnancy because they did not experienced previous contacts with parasites expressing antigens associated to placental malaria [4], [13]. On the other hand, studies on the infection impact in immune pregnant mice are also needed and we are building other experimental protocols that model the human disease observed in epidemiological conditions of high malaria transmission rate and high maternal pre-immunition.

Pregnant mice were more susceptible to *P. berghei-*GFP infection as compared to non-pregnant mice as they experienced faster increase in parasitemia and earlier death by hyperparasitemia (Figure 1). A significant proportion of the infected pregnant females most of the times abort, or even die before parturition, and no progeny is observed (Table 1). Maternal pre-immunition has been considered crucial for the disease severity, however hormonal changes and immuno-depression could have an important contribution to the observed increased susceptibility to infection during pregnancy [42]–[46]. Polyclonal activation associated with splenomegaly, hypergammaglobulinemia and immune-depression is commonly observed in *Plasmodium* infections. The spleen is a major site for *Plasmodium* clearance and its increased weight may represent a sign of activation status of the immune system revealing the presence of a current infection [47]. It is remarkable that the spleen weight of infected pregnant females was increased 5 fold when compared to the uninfected controls (Table 2), possibly representing an exacerbated activation of the maternal immune system by *P. berghei-*GFP infection during pregnancy.

Human placenta pathology associated with *P. falciparum* infection includes the following features: local parasitemia, malarial pigment deposits, excess of fibrinoid deposits, syncytiotrophoblast necrosis, trophoblast basement membrane thickening and macrophage infiltration [48].

We noted that iRBC were in intimate contact with placental tissue components and that hemozoin was widely spread in maternal blood spaces of infected placentas. It has been argued that hemozoin accumulates in tissue and within macrophages, remaining for several months after parasite clearance, leading to placental function impairment and favoring immunodepression [49], [50]. We hypothesize that accumulation of *P. berghei*-GFP iRBC in the placenta may evoke the inflammatory response that resembles the placental malaria pathology attributed to *P. falciparum*.

In response to the presence of iRBC, activated placental macrophages could induce placental damage, through releasing inflammatory cytokines such as TNF-α [51]. Hofbauer cells, placental resident macrophages, can be stimulated by placental parasites to produce β-chemokines that are chemotactic for monocytes/macrophages. This is in line with our observation that the chemokine MIP-1 alpha was upregulated in infected placentas. Such type of inflammatory triggering would explain the observed recruitment of a mononuclear infiltrate that predominate in maternal blood spaces of the labyrinthine zone. Activated macrophages could process and present antigens to maternal lymphocytes that, in turn, produce inflammatory cytokines, helping the parasite elimination [52], [53].

*P. berghei*-GFP infected placentas showed general tissue architecture disorganization with prominent thickening of the syncytiotrophoblast. This may result in part from fibrosis which has been proposed to arise from the reparative process stimulated by the response to infection [54]. It has been proposed that fibrinoid deposition confers fetus protection from maternal immune responses [55], [56] possibly concealing fetal antigens from the maternal immune system [55]. However, extensive fibrinoid necrosis and fibrinoid deposition are abnormal and typical of malaria infected placentas [51]. In our experimental model we consistently found fibrinoid lesions that were restricted to the maternal placental regions, either covering extensive areas or having focal distribution.

A striking pathological finding in *P. berghei*-GFP infected placentas was the reduction of blood sinusoids space (Figure 7), which is attributable to placental tissue thickening that presumably compressed available blood vascular space. The blood vascular area was reduced by 32% as compared to non-infected placentas implying an important placental blood volume reduction.

The effects of malaria on the newborn status are believed to be caused by placental insufficiency [57]. In fact, blood flow restrictions in infected placentas correlate well with fetal and postnatal development impairments observed in progeny from pregnant females infected with *P. berghei*-GFP (Figure 2). It is known that babies born to anemic mothers have low iron stores and are more likely to develop anemia [58]. Malaria in pregnancy is frequently associated with infant anemia and, consequently, child development and survival are at risk [59], [60]. Pathologic disorders may modify the intrinsic respiratory capacity of the placenta at any given gestational age, due to fibrinoid deposition that impairs gas diffusion, and to thickening that reduce the capillary area for exchange and the placental blood flow [61]. Consequently these placental changes caused by malaria will lead to insufficient *in uterus* hemoglobin/iron/oxygen availability and nutrients supply associated with an excess number of fetal circulating erythroblasts, which was consistent with our findings (Figure 5D). Although we could not detect a significant increase in Hypoxia-Inducible Factor-1alpha gene expression in infected placentas (data not shown), it is likely that tissue stress responses induced by the alterations in placenta blood circulation could play a role in placental malaria physio-pathology. PAM detrimental effects in mice offspring are somewhat diverse. Some fetuses survived, others were underweight and several died inside the uterus (Table 1 and Figure 3A). We observed lower birth weight in the newborns from mothers infected during pregnancy when compared to the ones from healthy mothers (*p* = 0.03). This weight difference was steadily maintained in post-natal life until weaning, even if newborn mice from infected mothers were fostered by non-infected mothers.

Although we have found important pathological changes in both basal and labyrinthine zones of mouse placenta, parasites and hemozoin were never visualized in the fetal circulation and positive parasitemia was never recorded in newborns from infected mothers. The absence of evidence for congenital infection, despite the presence of numerous iRBC in the placental maternal blood, points to the efficacy of the placental trophoblastic layer to block parasite traversing to fetal blood. The mechanism by which the trophoblastic cells prevent fetal infection is poorly understood, but several trophoblast defense mechanisms have been proposed [62]–[64]. These cells are not only able to perform phagocytosis *in vivo* and *in vitro* but, when properly stimulated via IFNγ, are also capable of increasing erythrophagocytosis activity [65].

The pattern of iRBC adherence in *P. falciparum* infected placentas remains controversial, but the main placental candidate receptors and their cognate parasite ligands participating in iRBC adhesion have been identified. Muthusamy *et al.* [40] established that the major natural receptors for iRBC adherence are localized primarily in the intervillous space of the placenta and at a lower extent on the syncytiotrophoblasts. It is known that iRBC bind different host molecules present in several organs, but CSA and HA have been suggested as mediators of parasite accumulation in the placenta [66]. CSA molecules from different sources differ in sulfatation patterns, with major consequences for iRBC adhesion [67], [68]. Highly sulfated forms can fail to support adhesion, whereas low-sulfated forms are optimal for binding [69], [70] and appear on the syncytiotrophoblast and in intervillous spaces [70]. HA has also been proposed as a candidate placental receptor [16], but its role in parasite adhesion is still debated [71], [72].

Our data show a significant reduction of placental adhesion when the iRBC were pre-incubated with of soluble HA (up to 90% reduction, compared to control) or CSA (up to 75% reduction, compared to control), indicating that these GAG can be important adhesion receptors in this model. Chondroitinase and hyaluronidase placental sections treatment also reduced adhesion considerably but did not completely abrogate binding. This is in line with *P. falciparum* previous studies demonstrating that the placental parasite populations do not adhere in a uniform manner to immobilized CSA or GAG receptors, implying that other factors may be confounding this interaction or other receptors are involved [73].

To demonstrate that the iRBC binding is receptor dependent, we treated iRBC with two different proteases and showed that binding properties of protease-treated iRBC were substantially inhibited, which was consistent with the involvement of surface membrane protein receptors in mediating adhesion. The *P. berghei-*GFP adhesion to CSA and HA indicates that iRBC could co-express ligands for different receptors, represented either by separate adhesive molecules or by different binding sites on a single molecule. Overall, the experimental data suggest that cytoadherence of *P. berghei-*GFP in the placenta may involve CSA and HA as receptors and raises the hypothesis that human and murine malaria in pregnancy have similar pathogenesis basis. Future efforts towards collecting in vivo evidence for iRBC binding will help to further establish CSA and HA as key pathogenesis mediators in placental malaria.

The phenotypic characterization of murine PAM here presented warrants that further research is needed to describe the pathogenesis mechanisms at cell and molecular levels. Although PfEMP1 orthologues were not yet found in *P.berghei*, our findings raise the interesting possibility that the receptors mediating adhesion in the mouse placenta could have in P.berghei iRBC cognate ligands that are related to PfEMP1. The prominent histological alterations in mouse placenta heavily infected with *P. berghei*-GFP resemble those described for acute *P. falciparum* malaria in humans [57], and future lines of research will clarify whether pathogenesis components, already identified in humans, have their counterpart in this model of severe malaria in pregnancy. Genetic manipulations of the model will offer opportunities to identify further host pathogenesis factors involved in placenta pathology. Thus, the experimental model here described may offer new opportunities to investigate the mechanisms implicated in placental malaria and in pregnancy outcome impairments, which are difficult to study in human subjects.
